# Loss of Dkk-1 in Osteocytes Mitigates Alveolar Bone Loss in Mice With Periodontitis

**DOI:** 10.3389/fimmu.2019.02924

**Published:** 2019-12-10

**Authors:** Paula Goes, Caio Dutra, Lennart Lösser, Lorenz C. Hofbauer, Martina Rauner, Sylvia Thiele

**Affiliations:** ^1^Division of Endocrinology, Diabetes and Bone Diseases, Department of Medicine III & Center for Healthy Aging, Technical University, Dresden, Germany; ^2^Department of Pathology and Legal Medicine, School of Medicine, Federal University of Ceará, Fortaleza, Brazil; ^3^Post-graduation Program in Morphofunctional Science, Department of Morphology, School of Medicine, Federal University of Ceará, Fortaleza, Brazil

**Keywords:** periodontitis, Dkk-1, osteocyte, inflammation, bone loss, osteoimmunology

## Abstract

**Background:** Periodontitis is a highly prevalent infection-triggered inflammatory disease that results in bone loss. Inflammation causes bone resorption by osteoclasts, and also by suppression of bone formation via increase of Dickkopf-1 (Dkk-1), an inhibitor of Wnt signaling. Here, we tested the hypothesis that osteocytic Dkk-1 is a key factor in the pathogenesis of periodontitis-induced alveolar bone loss (ABL).

**Methods:** Twelve-week-old female mice with a constitutive deletion of Dkk-1 specifically in osteocytes (Dkk-1^fl/fl^;Dmp1:Cre) were subjected to experimental periodontitis (EP). Cre-negative littermates served as controls. EP was induced by placing a ligature around the upper 2nd left molar, the contralateral side was used as control. Mice were killed after 11 days and maxillae removed for micro-CT and histological analyses. The mRNA expression of Dkk-1, Runx2, Osteocalcin, OPG, RANKL, RANKL/OPG ratio, LEF-1, and TCF-7 were assessed in maxillae, while mRNA expressions of TNF and IL-1 were evaluated on gingiva using real-time PCR. Blood samples were collected for Dkk-1, CTX, and P1NP measurement by ELISA.

**Results:** The deletion of Dkk-1 in osteocytes prevented ABL in mice with EP, compared to Cre-negative control mice with EP. Micro-CT analysis showed a significant reduction of bone loss (−28.5%) in EP Dkk-1^fl/fl^;Dmp1:Cre-positive mice compared to their littermate controls. These mice showed a greater alveolar bone volume, bone mineral density, trabecular number, and trabecular thickness after EP when compared to the Cre-negative controls. The local expression in maxillae as well as the serum levels of Dkk-1 were reduced in Dkk-1^fl/fl^;Dmp1:Cre-positive mice with EP. The transgenic mice submitted to EP showed increase of P1NP and reduction of CTX-I serum levels, and increase of TCF-7 expression. Histological analysis displayed less inflammatory infiltrates, a reduction of TNF and IL-1 expressions in the gingiva and fewer osteoclasts in Cre-positive animals with EP. Moreover, in mice with EP, the osteocytic deletion of Dkk-1 enhanced bone formation due to increased expressions of Runx2 and Osteocalcin and decreased expression of RANKL in maxillae.

**Conclusion:** In summary, Dkk-1 derived from osteocytes plays a crucial role in ABL in periodontitis.

## Introduction

Alveolar bone loss and connective tissue destruction are the characteristic clinical hallmarks of periodontitis, which is a highly prevalent and infectious-inflammatory disease, the second major cause of tooth loss worldwide (1). Periodontitis is mainly initiated by an oral biofilm. However, its development and progression is closely related to the exacerbated host response, which plays an important role on tissue breakdown (2).

It is known that inflammatory cytokines for example, TNF and IL-1beta, play an important role in periodontitis, inducing bone loss by promoting the expression of receptor activator of nuclear factor kappa-B ligand (RANKL) in other cells such as T cells and fibroblasts, favoring osteoclastogenesis (3). Recently, it was reported that osteocytes are also major producers of RANKL (4).

Despite the well-known RANK-RANKL axis, other pathways including the Wnt signaling have also been implicated in the process of bone loss (5). Wnt signaling is a crucial developmental pathway, and Dickkopf-1 (Dkk-1) is an important secreted inhibitor of Wnt signaling. Dkk-1 is expressed in various organs and by several cell types, although osteoprogenitors seems to contribute mostly to systemic Dkk-1 levels (6). It binds to lipoprotein receptor-related protein (LRP) 5/6 receptor blocking the interaction with Wnt proteins and leading to beta-catenin degradation. In bone tissue the lack of translocation of beta-catenin into the nucleus impairs the activation of osteoblast-related genes (Runx2, osteocalcin, and osteoprotegerin), leading to reduced osteoblastogenesis and low bone mass (7).

Besides bone homeostasis, Dkk-1 may play an important role in pathological bone loss (8) since inflammatory mediators induce Dkk-1 production (9, 10). It has been previously reported by our group and others, that during periodontitis, there is an increase of Dkk-1 in the periodontal tissue (11–13). However, to date, there is no substantial evidence regarding the actual origin and role of Dkk-1 in the bone loss related to periodontitis. Therefore, this study shows for the first time that Dkk-1 derived from osteocytes plays an essential role on periodontal bone loss.

## Materials and Methods

### Animal Selection

The experiments were performed on female young adult mice (12 weeks old) osteocyte-specific deletion of Dkk-1 (Dkk-1^fl/fl^;Dmp1:Cre) in a transgenic mouse line (C57BL/6 background), which were previously described (6). Respective Cre-negative littermates were used as controls. All mice were genotyped using standard PCR protocols.

Mice were maintained in groups of up to four animals per cage, weighing 20–25 g, and were kept in a dark cycle of 12:12 h at room temperature in filter top cages with cardboard houses as enrichment. Mice were randomly assigned to treatment groups and the subsequent analyses were performed in a blinded-fashion. All invasive procedures were approved by the Medical Faculty of the Technische Universität Dresden and the Landesdirektion Sachsen.

A power calculation was performed to determine the sample size. The animal was considered the study unit. The sample size was determined to provide 80% power to recognize a significant difference of 20% among groups and the standard deviation of 15% with a 95% confidence interval (*p* = 0.05), considering the change in alveolar bone loss (ABL) as the primary outcome variable. Therefore, a sample size of at least six mice per group was required.

### Experimental Periodontitis

After 2 weeks of acclimation to the laboratory environment, the mice were subjected to experimental periodontitis (EP). For that, the mice were anesthetized with ketamine (100 mg/kg body weight) and xylazine (10 mg/kg body weight) intraperitoneally. Following, all the animals received a sterile polyacrylamide ligature (6–0) around the cervical area of their maxillary left second molars (14, 15). After 11 days, all mice were euthanized. The contralateral right side was used as the unligated control.

### Assessment of Alveolar Bone Microarchitecture

For μCT measurements the maxillae were analyzed *ex vivo* (vivaCT40, ScancoMedical, Switzerland) with an isotropic voxel size of 10.5 μm (70 kVp, 114 μA, 200 ms integration time). Initially, the 3D reconstruction was performed and the measurement of ABL, in the buccal side, was performed using ImageJ software (National Institutes of Health, Washington, DC, USA). For that, the area between the cementum-enamel junction until the reminiscent bone border from left and right sides of the maxillae were used. For volumetric analyses 20 slices from the second molar were selected. Bone volume (BV/TV), bone mineral density (BMD), trabecular number (Tb.N), and trabecular thickness (Tb.Th) were assessed (16). All micro-CT analyses were performed by one blinded and calibrated examiner.

### Bone Histology and Histomorphometry

The maxillae were removed and fixed in 4% PBS-buffered paraformaldehyde for 48 h. Thereafter samples were demineralized using EDTA solution (Osteosoft®, Merck, Darmstadt, Germany). After that, the specimens were dehydrated and embedded in paraffin. Serial sections of 2 μm thickness were obtained in a mesio-distal direction. The sections were stained with hematoxylin and eosin and tartrate-resistant acid phosphatase (TRAP). Hematoxylin and eosin slides were performed to evaluate periodontal architecture and inflammatory status in the area between the first and second molars, using scores varying from 0 to 3 according to the intensity of findings, as follows: Score 0: absence or only discrete cellular infiltration, few osteoclasts, preserved alveolar process, and cementum; Score 1: moderate cellular infiltration, presence of some osteoclasts, some but minor alveolar process resorption and intact cementum; Score 2: severe cellular infiltration, large number of osteoclasts, accentuated degradation of the alveolar process, and partial destruction of cementum; Score 3: severe cellular infiltrate, total destruction of alveolar process, and cementum (17). Hematoxylin and eosin staining was also used to assess the number of osteoblasts per bone perimeter (N.Ob/B.Pm). Osteoblasts were characterized by its well-known cuboidal morphology and location over bone surface. The TRAP staining was performed to assess the number of osteoclasts per bone perimeter (N.Oc/B.Pm) using the Osteomeasure® software (OsteoMetrics, Atlanta, Georgia, USA) (18).

### RNA Isolation and Quantitative PCR

RNA was extracted from maxillae as well as gingiva of Dkk-1^fl/fl^;Dmp1:Cre mice by crushing them in liquid nitrogen and collecting the powder in Trifast (Peqlab, Germany). RNA isolation was performed according to the manufacturer's protocol. Five hundred nanograms of RNA were reverse transcribed using Superscript II (Invitrogen) and subsequently used for SYBR green-based real-time PCR (ABI 7500 Fast; Applied Biosystems). The primer sequences were: ß-actin s: ATCTGGCACCACACCTTCTT, ß-actin as: GGGGTGTTGAAGGTCTCAAA; Dkk1 s: GAGGGGAAATTGAGGAAAGC, Dkk1 as: AGCCTTCTTGTCCTTTGGTG, Runx2 s: CCCAGCCACCTTTACCTACA, Runx2 as: TATGGAGTGCTGCTGGTCTG, OCN s: GCGCTCTGTCTCTCTGACCT,OCN as: ACCTTATTGCCCTCCTGCTT, OPG s: CCTTGCCCTGACCACTCTTA, OPG as: ACACTGGGCTGCAATACACA, RANKL s: CCGAGACTACGGCAAGTACC, RANKL as: GCGCTCGAAAGTACAGGAAC, IL-1ß s: ACAAGGAGAACCAAGCAACG, IL-1ß as: GCCGTCTTTCATTACACAGG, TNF s: CCTCTTCTCATTCCTGCTTGTG, TNF as: CACTTGGTGGTTTGCTACGAC, LEF1 s: CAAATAAAGTGCCCGTGGTG, LEF1 as: TCGTCGCTGTAGGTGATGAG, TCF7 s: GGACATCAGCCAGAAGCAAG, TCF7 as: GGACAGGGGGTAGAGAGGAG. PCR conditions used were: 50°C for 2 min and 95°C for 10 min followed by 40 cycles with 95°C for 15 s and 60°C for 1 min. The melting curve was assessed by the 95°C for 15 s, 60°C for 1 min, and 95°C for 30 s. The results were calculated using the ΔΔCT method and are presented as x-fold increase relative to beta-actin (18).

### Serum Analysis

Blood was taken via heart punctuation and serum was collected after 10 min centrifugation at 400 × g. Dkk-1, C-terminal telopeptide (CTX) and type 1 procollagen amino-terminal-propeptide (P1NP) were measured using an immunoassay kit (Dkk1: R&D Systems, USA; CTX and P1NP: Immundiagnostik Systems, Germany) according to the manufacturer's protocols.

### Statistical Analysis

The data are presented as means ± standard errors of the mean or as median (range), when appropriate. Normality and homoscedasticity of the data were verified. ANOVA followed by the Bonferroni test were used to compare the means, and Kruskal–Wallis and Dunn's tests were used to compare the medians. The significance level was set at 5% in all tests. All calculations were performed using Prism 5 (GraphPad Software Inc., San Diego, CA, USA). All protocols and analyses were performed by blinded and calibrated examiners.

## Results

### Osteocytic Deletion of Dkk-1 Prevents Periodontal Bone Loss

**Figure 1:**
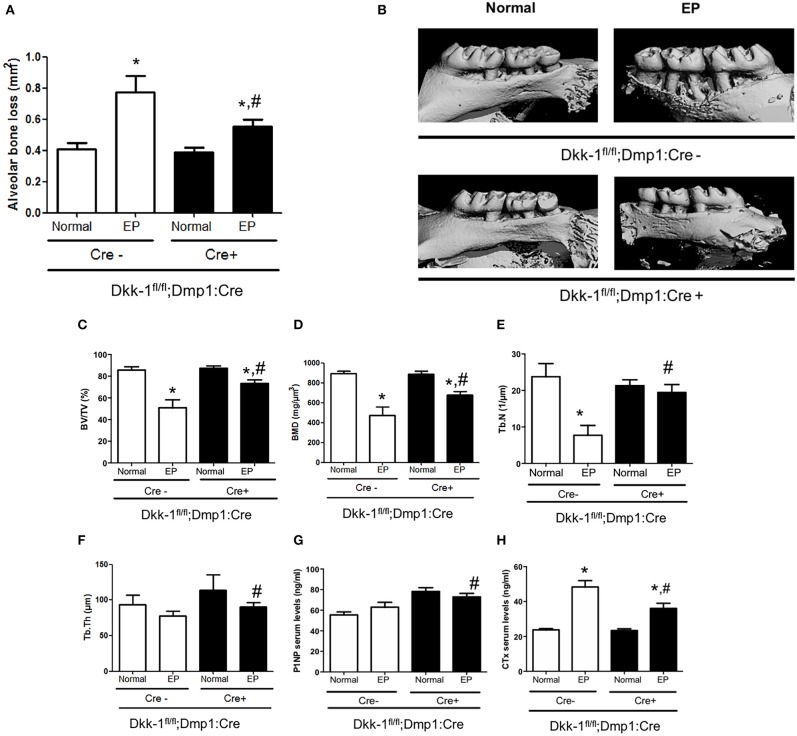
Lack of Dkk-1 in osteocytes prevents periodontal bone loss. Maxillae of 12-week-old female Dkk1fl/fl;Dmp1:Cre-positive and -negative mice were analyzed by μCT. **(A)** Alveolar bone loss, **(B)** representative 3D reconstruction of hemimaxillae with and without ligature in buccal view, **(C)** trabecular bone volume per total volume (BV/TV), **(D)** bone mineral density, **(E)** trabecular number (Tb.N), and **(F)** trabecular thickness (Tb.Th) of hemimaxillae. **(G)** Serum levels of procollagen type 1 aminoterminal propeptide (P1NP) and **(H)** carboxy-terminal collagen cross-links (CTX-I) were assessed using commercially available ELISAs. Data represent the mean ± SEM of at least six animals per group. Statistical analyses were performed by ANOVA followed by the Bonferroni test. *P < 0.05 vs. respective normal hemimaxillae; ^#^*P* < 0.05 vs. EP Cre-negative control.

In Cre-negative mice, EP caused significant alveolar bone loss (Figures 1A,B) as well as reduction of bone volume (−40.6%) (Figure 1C), bone mineral density (−46.7%) (Figure 1D) and trabecular number (−67.6%) (Figure 1E), when compared to the control site. Loss of Dkk-1 derived from osteocytes, however, resulted in less alveolar bone loss (−28.5%) in mice subjected to EP compared to the EP Cre-negative group (Figures 1A,B). In line with these results, also bone volume (−24.6%) (Figure 1C), bone mineral density (−23.3%) (Figure 1D), trabecular number (−58.8%) (Figure 1E), and trabecular thickness (−3.1%) (Figure 1F) showed milder reductions compared to the Cre-positive mice.

We also evaluated the serum bone turnover markers and found high levels of CTX after EP induction in Dkk-1^fl/fl^;Dmp1:Cre-negative mice (*p* < 0.05) (Figure 1H). In Dkk-1^fl/fl^;Dmp1:Cre-positive animals, CTX levels were significantly reduced together with increased P1NP serum levels (Figure 1G).

### Osteocytic Deletion of Dkk-1 Activated Wnt Signaling During Periodontitis

**Figure 2:**
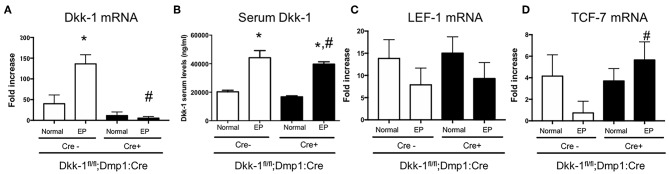
Lack of Dkk-1 in osteocytes activated Wnt signaling during periodontitis. **(A)** Real-time PCR analysis was performed for Dickkopf-1 (Dkk-1) in hemimaxillary bone tissue. **(B)** Serum levels of Dkk-1 were assessed using commercially available ELISA. **(A)** Real-time PCR analysis was performed for **(C)** LEF-1 and **(D)** TCF-1 were also performed in hemimaxillary bone tissue. Data represent the mean ± SEM of at least six animals per group. Statistical analyses were performed by ANOVA followed by the Bonferroni test. *P < 0.05 vs. respective normal hemimaxillae; ^#^*P* < 0.05 vs. EP Cre-negative control.

Dkk-1 gene expression in maxillae (Figure 2A) as well as Dkk-1 serum levels (Figure 2B) were also investigated. EP significantly increased Dkk-1 gene expression in maxillae only in Cre-negative, but not Cre-positive mice. No differences were seen in the serum levels of Dkk-1 between Cre-negative and Cre-positive mice submitted to periodontitis. The expression of Wnt target genes LEF-1 (Figure 2C) and TCF-7 (Figure 2D) were evaluated. Periodontitis tended to reduce the expression of LEF-1 and drastically reduced TCF-7 expression in wildtype littermates. However, in Dkk-1 conditional knock-out mice with periodontitis, TCF-7 expression remained at control level (Figure 2D). There was no change on the expression of LEF-1 (Figure 2C).

### Osteocytic Deletion of Dkk-1 Modulates Inflammation and Increases Osteoblast Activity During Periodontitis

**Figure 3:**
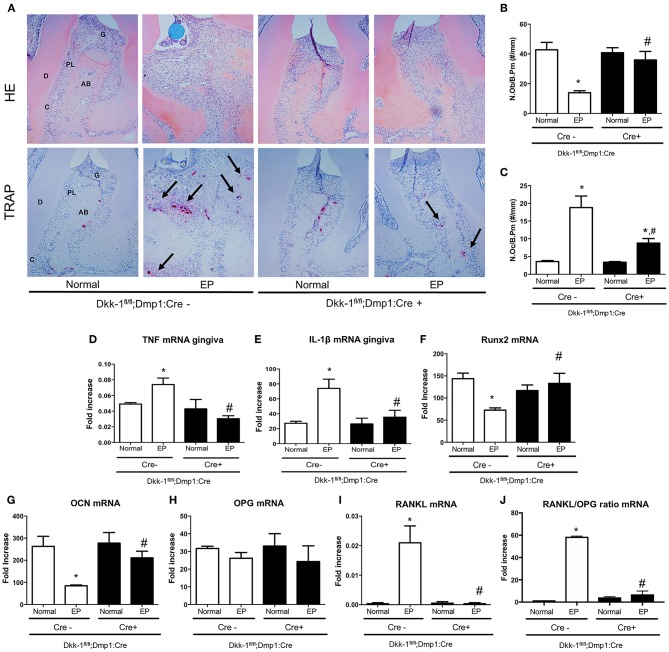
Lack of Dkk-1 in osteocytes modulates inflammation and enhances bone formation. **(A)** H&E and TRAP staining of hemimaxillae 11 days after periodontitis induction were analyzed to assess **(B)** number of osteoblasts (N.Ob./B.Pm.) and **(C)** number of osteoclasts (N.Oc./B.Pm.). Real-time PCR analysis was performed for **(D)** TNF and **(E)** IL-1β in gingiva as well as for **(F)** runt-related transcription factor 2 (Runx2), **(G)** osteocalcin (OCN), **(H)** osteoprotegerin (OPG), **(I)** receptor activator of nuclear factor-kB ligand (RANKL), and **(J)** RANKL/OPG ratio, in hemimaxillary bone tissue. Data represent the mean ± SEM of at least six animals per group. Statistical analysis was performed by ANOVA followed by the Bonferroni test. *P < 0.05 vs. respective normal hemimaxillae; ^#^*P* < 0.05 vs. EP Cre-negative control.

The osteocytic deletion of Dkk-1 maintained the periodontium architecture in mice submitted to EP when compared to the Cre-negative group (Figure 3A). In the normal maxillae of either Cre-negative or Cre-positive mice, it is possible to observe the normal organization of the periodontal tissue [0 (0–0)]. However, EP in Cre-negative animals provoked the great amount of inflammatory infiltrate on the gingival tissue, as well as bone and cementum resorption, marked by an increase of osteoclasts [3 (2–3)] (Figure 3C), which was statistically significant when compared to the Cre-negative control. All these histological findings were mitigated in the mice with osteocytic deletion of Dkk-1 submitted to EP [1 (1–2)] (Figure 3A) (*p* < 0.05). Furthermore, the number of osteoblasts significantly increased (Figure 3B) in these animals.

The effect of osteocytic deletion of Dkk-1 in the periodontal inflammation was confirmed by the downregulation of TNF (Figure 3D) and IL-1β (Figure 3E) gene expression in the gingiva when compared to the Cre-negative group, both submitted to EP (*p* < 0.05).

The analysis of gene expression in maxillae showed that periodontitis caused a significant decrease of Runx2 (Figure 3F) and OCN (Figure 3G), as well as an increase of RANKL (Figure 3I) in Cre-negative control mice. However, when Dkk-1 derived from osteocytes was deleted, an increase of Runx2 and OCN expression was observed (p < 0.05). While OPG expression was not affected (Figure 3H), expression of RANKL in the Dkk-1^fl/fl^;Dmp1:Cre-positive mice with EP was significantly decreased compared to the Cre-negative control mice. Thus, the osteocytic deletion of Dkk-1 reduced the RANKL/OPG ratio (Figure 3J), which may explain the reduced activation of osteoclasts.

## Discussion

In this study, we show that the deletion of Dkk-1 derived from osteocytes plays an important role in the pathogenesis of periodontal bone loss. The osteocytic Dkk-1 deletion reduced bone loss, mitigated inflammation, and enhanced bone formation in mice submitted to ligature-induced periodontitis.

Importantly, the osteocytic-specific deletion of Dkk-1 prevented EP-induced bone loss. The Dmp1 promoter has been shown to target osteocytes, mature osteoblasts, and occasional bone lining cells (4, 19), even though also non-specific deletions have been observed in muscle, intestine, and brain (18). Nonetheless, these mice have been characterized well and show a significant reduction of Dkk-1 in cortical bone tissue, which mostly contains osteocytes, while no decrease is observed systemically (6). This study now further shows that Dkk-1 mRNA levels are reduced in the maxillae of Dkk-1^fl/fl^;Dmp1:Cre-positive mice and that osteocytes mostly contribute to EP-induced Dkk-1 levels in the maxilla, as this increase was absent in Dkk-1^fl/fl^;Dmp1:Cre-positive mice. Thus, this study supports the previous observation from our group that local, but not systemic Dkk-1 levels are critical to determine bone loss.

TCF/LEF are transcription factors for β-catenin expressed in the nucleus, that mediate the canonical Wnt signaling in several cell types (20). An increase on TCF expression was observed in mice submitted to EP with osteocytic deletion of Dkk-1. Different from LEF, TCF was detected in prechondrocytes in the palate, nasal bone, occipital bone, vertebrae, ribs, and jaws during mouse embryo (21). Moreover, it has been reported that Tcf^−/−^ mice have an increased osteoclast number and function without any change in osteoblast number of function (22), and also showed accelerated bone resorption (23). Consistent with our results, Shin et al. (24) reported that the activation of beta-catenin/TCF decreased the expression of RANKL while mRNA level of OPG was unchanged. However, whether RANKL is a direct target of the beta-catenin/TCF pathway is not clear and requires further work.

Osteocytes are now considered master regulators of osteoblast and osteoclast function by connecting with them via their dendritic processes (25). In fact, osteocytic deletion of Dkk-1 increased osteoblast numbers and reduced osteoclast parameters in the maxillae of mice with EP, suggesting that Dkk-1 derived from osteocytes is a critical factor in the communication with osteoblasts and osteoclasts during EP. Moreover, P1NP levels were increased, while CTX levels were decreased in osteocyte-specific Dkk-1 knock-out mice with EP. Mechanistically, this may be derived from increased Wnt signaling in osteoblasts, which may activate bone-related target genes. Our results showed an increase of Runx2 expression, which is a key transcriptional modulator of osteoblast differentiation (26, 27). Moreover, osteocalcin expression was increased, a major non-collagenous protein that is important for both, the biological and mechanical functions of bone (28). Finally, there was a significant reduction of RANKL expression in the maxillae, leading to a reduced RANKL/OPG ratio, which may account for the reduced number of osteoclasts. Similar reductions of the RANKL/OPG ratio were also found in other studies using Dkk-1-deficient mice (18). Taken together, these findings indicate the osteocyte-derived Dkk-1 plays an important role in modulating osteoblast and osteoclast function in EP.

Inflammation is a major trigger for bone loss, and our data show that the lack of Dkk-1 in osteocytes during periodontitis resulted in less inflammatory infiltrates. Therefore, the immunomodulation seen in this study may indicate that osteocyte-derived Dkk-1 is necessary for the initiation of the inflammatory process. This was confirmed by the reduced expression of TNF and IL-1β in the gingiva of animals with specific-deletion of Dkk-1 in osteocytes submitted to periodontitis. TNF and IL-1β are well-known key cytokines for periodontal disease (29). Furthermore, Dkk1 produced by platelets has been shown to control neutrophil invasion in acute lung inflammation via modulating ICAM expression (30) and the inflammatory interaction with endothelial cells during atherosclerosis (31). This, it could be envisaged that also osteocyte-produced Dkk1 alters the alveolar bone microenvironment in such a way to promote immune cell attraction and subsequent inflammatory reactions. Further, studies have shown an immunomodulation role of Dkk-1 in cancer immune surveillance (32) or promoting pathological chronic type 2 inflammation (33). Recently, it was demonstrated that Dkk-1 is uniquely expressed in Foxp3^+^ Treg cells to inhibit T-cell-mediated autoimmune colitis as a membrane-bound form (34). However, despite the eminent role of osteocyte derived Dkk-1 on inflammation, the exact mechanism of the immunomodulatory property of Dkk-1 deserves further investigations.

In summary, within the limits of this study, our findings emphasize the role of osteocytes in periodontitis, demonstrating for the first time that Dkk-1 secreted by osteocytes is essential for periodontal bone loss. These findings may contribute to a better understanding how osteocytes act on inflammatory bone loss and may also be important for the view of Dkk-1 as a mechanism that could be targeted in the future in bone diseases.

## Data Availability Statement

All datasets generated for this study are included in the article/supplementary material.

## Ethics Statement

The animal study was reviewed and approved by the Institutional Animal Care Committee and the Landesdirektion Sachsen.

## Author Contributions

PG, MR, and ST designed the study. PG and ST induced periodontitis and performed all the assays. CD and LL performed the molecular biology assay. LH and MR supervised the study. All authors contributed to the interpretation of the results.

### Conflict of Interest

The authors declare that the research was conducted in the absence of any commercial or financial relationships that could be construed as a potential conflict of interest.
